# Ethical and epistemological considerations in applying moral injury (MI) concepts to refugee populations

**DOI:** 10.1007/s11019-024-10237-4

**Published:** 2024-11-30

**Authors:** Roghieh Dehghan

**Affiliations:** Centre for Multidisciplinary and Intercultural Inquiry, University College London, London, UK

**Keywords:** Moral injury, Trauma, Refugees, Epistemic injustice, Ethics

## Abstract

Empirical research on moral injury (MI) has rapidly evolved since 2009. Originally developed to address the moral dimensions of traumatic experiences among US veterans, MI has also found application in the context of traumatized refugees. This paper delves into the ethical and epistemological questions that arise when applying a concept originally rooted in a qualitatively distinct experience and a demographically different population to refugees. It is argued that the prevailing clinical and psychological conceptualization of MI may not adequately accommodate the unique needs and experiences of refugee populations. This examination underscores the imperative of conceptualizing to better serve the ethical and epistemic demands of refugee communities.

## Introduction

Over the past decade, there has been a growing academic interest in moral injury (MI), a notion developed within modern trauma discourse that refers to the impact of the violation of deeply held moral beliefs on individuals (Litz et al. 2009). MI was introduced as a construct to shed light on the psychological struggles faced by US soldiers, but it is now used in a much wider range of contexts (Ricardson et al. 2020).

One field where this concept has gained substantial ground is refugee mental health research. Given that refugees are ten times more likely to report post-traumatic stress disorder (PTSD) than the general population in Western countries (Fazel et al. 2005; Porter & Haslam 2005; Zimmerman 2011), it does not come as a surprise that MI, a notion referring to the violation of deeply held moral beliefs (Litz et al. 2009) that was designed in response to the perceived weakness of PTSD, has also been expanded to this particular cohort of trauma survivors.

Since the MI construct, as developed for military personnel, is also being applied to refugees, it is essential to investigate the nature of MI in more depth. Accordingly, this paper has been divided into three parts: the first section will map the history and current conceptualization of MI in the clinical discourse dominating the trauma discourse, highlighting some of the limitations of the conception of ‘morality’ presupposed in this literature. The second part will expand on this discussion by presenting a literature review on MI in refugees. In the final section, the discussion is expanded through the introduction of alternative models of morality that are attentive to the moral responses of individuals who experience the enactment of others’ moral principles and judgements on themselves. These models consider morality as ‘constructed and produced between’ individuals (Walker 2007, 9), highlight the embodied experiences of victims of moral transgressions (Bernstein 2015), and focus on the interpersonal and relational dimensions of morality (Wiinikka-Lydon 2019). These models will then lay the groundwork for addressing the ethical and epistemological challenges inherent in extending the psychological discourse on MI to refugee populations.

## Background: moral injury

The conceptualization of suffering is influenced by societal processes and the cultural organization of illness as an expert domain. (Papadopoulos 2020, 7). Psychological disciplines are often regarded as uniquely positioned to provide the epistemological tools necessary to articulate, quantify, and qualify the implications of human adversity. Given that trauma has become a primary testament to human suffering over the 20th century (Fassin & Rechtman 2009), it is unsurprising that these disciplines have also spearheaded the discourse on MI.

Philosopher Wiinikka-Lydon (2022) distinguishes between the first wave and the second wave of MI research. The first wave dates back to the 1990s when the term was coined by Jonathan Shay, a psychiatrist at the Department of Veterans Affairs in Boston, Massachusetts. In his book Achilles in Vietnam (Shay, 1994), Shay drew on Homer’s Iliad and Odyssey to describe MI in terms of betrayal by military authority. In his view, MI is ‘betrayal of what’s right by someone who holds legitimate authority (e.g., in the military—a leader) in a high stakes situation’ (Shay, 2014, 183). However, the notion did not really take off until psychologist Bert Litz and colleagues (2009) published an article on MI that did not refer to Shay’s work at all. Of the 124 studies in Richardson et al.’s (2020) review, 96 cite the definition of MI by Litz et al. (2009). In their definition, MI is ‘the lasting psychological, biological, spiritual, behavioral, and social impact of perpetrating, failing to prevent, or bearing witness to acts that transgress deeply held moral beliefs and expectations’ (Litz et al. 2009, 697).

Litz et al.’s (2009) seminal paper caused an exponential rise in research on MI. This second wave of MI is believed to be a response to modern warfare and guerrilla tactics that obscure the distinction between civilians and enemy combatants in ways that lead to unique moral dilemmas for soldiers (Farnsworth et al. 2017; Carey et al. 2016). What is more, the long wars in Iraq and Afghanistan and the difficulties of reintegrating into civilian life compound veterans’ psychological struggles and make it more difficult for them to reintegrate into civilian life (Jones 2020).

The reintegration struggle of military staff in the US led to MI being used to refer to agent-related experiences. Psychologists tend to want morality to be something that is measurable and subjectively affirmed by individuals. Hence, the psychological perspective of MI, which is mainly used by clinicians in trauma discourse, adopts an individual, cognitive, and behavioral notion of morality. Moral decisions, reasoning, and judgment of autonomous individuals are salient features in this approach. By focusing on a person’s beliefs and intra-psychic processes, morality becomes a property of an autonomous and rational individual. In this model, morality’s primary themes are an individual’s judgement of *good* versus *bad* concerning self or other, and *rightness* versus *wrongness* of specific actions.

The clinical position distinguishes between organizational/betrayal-based and individual-based MI. Even though most clinical work builds on Litz et al.’s (2009), they still associate betrayal with MI, the central feature of Shay’s (2014) model. In Shay’s (2014) view, for MI to occur, the betrayal takes place by authority figures in a high-stakes environment. However, researchers have expanded Shay’s concept of betrayal to include situations that are not high-stakes, like poor support by political leaders or public judgment (Eikenaar 2022; Molendijk 2018). Similarly, Jinkerson (2016) considers the term authority to suggest a trusted figure and therefore takes betrayal to be systemic, interpersonal, and intrapersonal, where one fails one’s own values.

The literature distinguishes between causes of MI, which are the potentially morally injurious events (PMIEs) and impacts of these events. PMIEs are thought to involve contextual variables such as organizational or leadership issues, situational factors (e.g., uncertain or high-risk environments), and actions like dehumanizing the enemy, extreme violence, and the loss of a comrade (Currier et al. 2020; Drescher et al. 2011). However, MI outcomes are distressing emotions and symptoms of MI (Mcewen et al. 2022).

The relationship between PMIEs, MI outcomes and mental health has been the focus of recent systematic reviews and meta-analyses (Williamson et al., 2018; McEwen et al., 2022). These studies illustrate statistically significant associations between PMIEs, MI, and mental health conditions such as PTSD, depression, and anxiety, with the largest effect size observed in the association between MI and PTSD. However, interpreting these findings requires caution due to various methodological issues. First, there is an overlap between events causing PTSD and those leading to MI. For instance, traumatic events central to a PTSD diagnosis can also be potentially morally injurious, and there is symptom overlap between PTSD and MI, particularly in areas such as cognition and mood. Second, there are concerns about the quality of studies in the aforementioned reviews, with most being cross-sectional and thus only able to establish association rather than causation. What is more, many MI scales conflate causes (PMIEs) and effects of MI, making it difficult to determine whether their scores reflect exposure to morally injurious events or the resulting distressing emotions and symptoms. Lastly, these reviews heavily relied on military populations, with 51 out of 59 papers in McEwen et al. (2022) and nine out of 13 articles in Williamson et al. (2018) focusing on such groups, predominantly from the United States.

Given the context of US soldiers, the core moral emotions that most empirical studies and MI scales focus on are predominantly shame, guilt, and anger. Moral emotions - self-focused (e.g., shame and pride) or other-focused (e.g., guilt and gratitude) - are expressions and representations of moral beliefs, an outcome of an individual’s perception and evaluation of moral properties. Moral emotions are described as adaptive (Farnsworth et al. 2017) or distorted (Jinkerson 2016). Since, in view of this clinical understanding, the purpose of morality is survival, emotions become secondary to fear so that the value of moral emotions and moral behaviors is seen in their function for survival, communication, and social cohesion (Drescher & Farnsworth 2020; Farnsworth et al. 2017; Litz et al. 2009).

While recently, the psychological framework of MI has also gained prominence in a wide range of other academic disciplines, there seems to be no consensus on the place of MI in trauma discourse. Some clinicians and scholars argue that MI fits under the rubric of PTSD but may still help to explain PTSD better and plan appropriate interventions (Phelps et al. 2022). Others assert that PTSD and MI are distinctive constructs, with differences in acquisition and expression (Currier et al. 2020). What is more, there are also social science and philosophical perspectives that consider MI a fundamentally different concept to PTSD, challenging psychiatric assumptions and the dominance of PTSD (Kinghorn 2020; Wiinikka-Lydon 2019). These alternative perspectives will be employed in this paper to critically evaluate the clinical model of MI as it is applied to refugees. However, prior to this evaluation, it is essential to examine how the contemporary discourse on MI within the trauma field has been integrated into discussions about refugee populations.

## Refugees and moral injury

A literature search on Scopus (undertaken in August 2022), one of the largest databases, for moral* injur* and refugee* in article title, abstract and keywords show that since 2015, there have been ten empirical studies conducted with ethnically and nationally heterogenous cohorts of refugees and asylum seekers residing either in Australia, Switzerland or the Netherlands. With only one exception, a qualitative semi-structured interview study (Passardi et al. 2022), all studies were mainly correlational, assessing the association between moral injury with common mental health outcomes (Nickerson et al. 2015, 2018, 2020; Hoffman et al. 2018, 2019; Hoffman and Nickerson, 2018; Spaaij et al. 2021; Mooren et al. 2022; McEwen and Jobson 2022a, b).

It is also important to note that psychometric tools for MI have not been validated in non-military population. Construct validity refers to an instrument’s ability to measure what it is intended to measure (Stone 2019). Initially, researchers used an adapted a psychometric tool grounded in their expert opinions and the experiences of military personnel. Colonel William P. Nash (2013), a military psychiatrist who served in Iraq, and Brett Litz (2009), a military psychologist, established the foundational scales and conceptualizations of MI. Their work, based on clinical practice and research with U.S. military personnel, led to the development of the Moral Injury Event Scale (MIES). However, these scales had not been evaluated in non-military cohorts (Coady et al., 2021). Nonetheless, Nickerson et al. (2015) adapted the MIES for use with refugees by focusing on moral transgressions by others rather than oneself. They administered a 6-item measure to 134 treatment-seeking refugees and asylum seekers from various nationalities in Switzerland.

Shortly after that, drawing directly from MIES (Nash et al. 2013), Hoffman and colleagues (2018) developed the Moral Injury Appraisal Scale (MIAS) for refugee population. The MIAS is a 9-item measure where 4 items are categorised under MI-other (MI-O) and 5 items under MI-self (MI-S) depending on whether the agent of moral transgression is reported as oneself or another person. Their results demonstrated that trauma exposure predicted MI, but found that betrayal, unlike in military population, was not a good fit for MI in refugees. They also confirmed that both MI-O and MI-S predicted anger and depression, but only MI-O had a positive correlation with PTSD.

Table 1 presents MIES (Nash et al. 2013) for military next to MIAS (Hoffman et al. 2018) for refugees. The critique that has been levelled to MIES concerning the conflation of exposure (PMIE) and outcome (moral injury), which renders conclusions about associations problematic, applies to MIAS too. It also is important to highlight that the change of the word from ‘event’ in MIES to ‘appraisal’ in MIAS is merely a semantic choice that reflects Hoffman et al.’s (2018) stress on the significance of appraisal in the impact of potentially morally injurious events (PMIE) rather than any meaningful modification to the actual items in the respective scales.

**Table 1:** 11-item MIES for military and 9-item MIAS for refugees

MIES (Nash et al. 2013)	MIAS (Hoffman et al. 2018)
I saw things that were morally wrong	I am troubled by morally wrong things done by other people
I am troubled by having witnessed others’ immoral acts	I am troubled because I saw other people do things that were morally wrong
I acted in ways that violated my own moral code or values	I am troubled because I heard about other people doing things that were morally wrong
I am troubled by having acted in ways that violated my own morals or values	I am troubled because other people have acted against important moral rules
I violated my own morals by failing to do something that I felt I should have done	I am troubled because I did things that were morally wrong
I feel betrayed by leaders who I once trusted	I am troubled by morally wrong things I have done
I feel betrayed by fellow service members who I once trusted	I went against my own morals by failing to do something I should have done
I feel betrayed by others outside the U.S. military who I once trusted	I am troubled because I did things that were morally wrong
I am troubled because I violated my morals by failing to do something that I felt I should have done	I am troubled because I acted in ways that went against my own moral code or values
I trust my leaders and fellow service members to always live up to their core values	
I trust myself to always live up to my own moral code	

While the first study that applied MIES (Nash et al. 2013) to refugees (Nickerson et al. 2015) found a strong association between moral injury and PTSD, depression, anger over and beyond post-migratory stressors and a dose-response relationship to trauma, the subsequent studies that used MIAS reported conflicting results in terms of an association between moral injury with trauma, on the one hand, and with mental health outcomes on the other hand. For example, in Nickerson et al’s (2018), trauma exposure at baseline predicted MI-O after 2–4 years, but not MI-S, and MI-O was associated with PTSD and depression at baseline but not long-term. In contrast, another longitudinal study (Nickerson et al. 2020) showed that MI-O predicted long-term PTSD symptom as well as sadness, anger, and shame.

Interestingly, Hoffman and colleagues (2019) have, following a recent study, critiqued their own scale (MIAS) by pointing out that MI-S may be irrelevant to refugee populations as their participants did not report moral violations committed solely by themselves but either MI-O or a combination of MI-O and MI-S (MI-O + S). Indeed, after distinguishing between MI-O and MI-O + S, moral injury profiles seem to be associated with greater psychopathology beyond the impact of pre-migration trauma.

One of the main issues is that the psychometric instruments used (MIES and MIAS) have not been adequately validated in refugee population. With construct validity under question, reliability could then equally imply that results are merely consistently reproduced by using the same invalid tools (Kleinman 1987). However, the most serious limitation of all these studies is that the parameters of the concept moral injury originated with military population - I will expand on this issue in the next section.

In short, MI indeed may be a promising framework for investigating refugee populations’ enduring but poorly understood psychological difficulties. However, the generalisability of a notion created for Western military context calls for critical reflection on the ethical and epistemological issues that arise when applying a concept rooted in military personnel to refugees.

### Ethical and epistemological issues of applying MI to refugees

Moral injury is a multifaceted concept that should be ‘interdisciplinary, intersectional, contextualized, and polyvocal’ to accurately reflect the complex moral realities that are ‘always socially located, partial, and related to our agendas and exercise of power in the world’ (Lettini 2020, 33). In light of this complexity, the problems posed by applying a psychological discourse on MI to refugees can be categorised as pistemic and ethical. Some of the relevant questions are: is it reasonable to assume that refugees are exposed to a similar kind of violence as the military population? Are there factors – considering citizenship, race, and culture - that may make refugees different from veterans, leading to alternative interpretations of study findings and, consequently, a distinct conceptualization of MI?

These concerns align with what Fricker (2007) terms hermeneutical injustice. Her work on epistemic injustice eloquently integrates epistemology and ethics, underscoring how knowledge is socially constructed and influenced by power dynamics that affect the recognition and validation of knowledge. Fricker (2007) highlights that the knowledge contributions of marginalized groups are often undervalued or dismissed. According to her, hermeneutical injustice arises when the experiences of marginalized groups are misunderstood or misrepresented due to a lack of adequate conceptual tools. This inadequacy results in inequalities in the conceptualization, representation, and interpretation of these groups’ experiences, rendering their experiences unintelligible.

While the extension of MI beyond military context was undertaken with the aim of providing a framework for addressing symptoms similar to those experienced by soldiers, the use of MI to theorize refugee experiences may still result in epistemic injustice, albeit unintentionally. I therefore argue that that the current conceptualization of MI, which parameters are based on experiences from cohorts culturally and experientially distinct from refugees, is both epistemically unsound and ethically problematic. I make the proposition that it is epistemically flawed to assume that conceptual models and psychometric scales that were developed to draw attention to the ‘humanity’ of perpetrators of violence (Litz & Kerig 2019, 41) work equally well for the victims of violence. For example, ‘humanity’ in the context of a commissioner of a moral transgression may be linked to notions such as self-forgiveness (Jones 2020), whereas for the victim, it might encompass moral demands for justice (Passardi et al. 2022). To fail to ground the normative understanding of morality, its key parameters, and the cognitive-affective responses to moral violations in the experience of refuges has epistemic and ethical implications.

From an epistemic perspective, the current framing of MI may overlook elements of trauma experience that are distinctive and crucial in refugees who endure a complex spectrum of potentially morally injurious events before, during, and after migration (Zimmerman 2011). For instance, Passardi and colleagues (2022) conducted in-depth interviews with 13 refugees in an offshore detention centre in Australia. In this pre-settlement phase, interviewees perceived themselves as placed outside a framework of moral and ethical obligations. Interestingly, their concept of moral transgressors extended beyond individuals to include the Australian government. The significant moral themes identified in their study - such as powerlessness, hopelessness, mistrust, dehumanization, and a loss of agency as well as belief in the benevolence of the world (ibid. 9) - are not adequately addressed by conventional psychometric tools for MI.

Besides, MI traditionally focuses on singular, morally injurious events from the past, akin to the experiences of soldiers returning from deployments in conflict zones like Iraq or Afghanistan. Yet, unlike a soldier who may leave a warzone and return home, migration is not a chapter that can be neatly closed; rather, it involves enduring and ongoing challenges that persist long after resettlement. Indeed, several studies demonstrate that post-migratory experiences - such as living difficulties and discrimination - significantly impact MI. MI in refugees encompasses trauma that extends beyond traditional boundaries, highlighting the need to address these persistent, evolving issues (Hoffman et al. 2019; McEwen et al. 2022; Nickerson et al. 2018).

If traumatized refugees and veterans are significantly different populations, then applying MI scales designed for one group on the other can compromise the validity of the studies so that their results may lead to mistaken assumptions about the individuals and the treatments they require. Simply translating questionnaires is not enough to ensure construct validity across diverse contexts, as validity encompasses semantic and cultural domains too. Cultural validity is about ensuring that an instrument measures what it says it measures with reference to truthfulness of a theory in a particular culture (Kleinman 1987). This is not something researchers can achieve through a mere translation of a questionnaire originally developed for a completely different, even contrasting, experiential cohort. There are terms and concepts that do not necessarily exist cross-culturally. Distress idioms are culturally loaded and local. For example, in our ongoing empirical study on the moral dimensions of trauma amongst Iranian refugee torture survivors in the UK, we have identified idioms that, when translated literally, would be rendered as ‘resistance’ and ‘humanity’. However, these terms carry distinct socio-cultural and historically situated meanings within Iranian culture—nuances that are frequently overlooked in Western-based psychological models of MI. Consequently, using invalid or inadequate tools in such contexts can lead to the development of concepts that are flawed at best and harmful at worst.

To capture the moral aspects of the situation of refugees, who often find themselves in the circumstances controlled by others, concepts related to an individual’s moral decision-making or self-transgression of rules and principles may be less relevant. Given the socio-political positioning and power dynamics affecting refugees, victim- or recipient-centered perspectives may be more appropriate for them (Bernstein 2015; Wiinikka-Lydon 2019). In fact, a perspective shift from perpetrator to victim testifies to different forms of suffering and draws attention to other moral emotions.

A profound insight into the world of a victim of moral violations is poignantly provided in the work of Jean Améry (1980), who recounts his torture in Auschwitz. His autobiographic book *At the Mind’s Limits* highlights how torture leads to severe dignitary harms, as the victim can ‘no longer feel at home in the world’, experiencing a permanent disconnection from humanity, where one’s fellow human beings are seen as ‘the antiman’ (ibid., 40). Améry demonstrates how being ‘de-humanized’, ‘de-intellectualized’, and robbed of the possibility of transcendence leave a lasting impact on the victim’s sense of self (ibid., 7–9).

[D]ifferently placed people know different things’ (Walker 2007, 7) and attend to varying aspects of their social worlds to interpret the moral dimensions of their experiences. This divergence in focus leads to the development of distinct epistemologies. While the MI discourse tends to refer to Greek mythologies and the warriors in Homer’s *Iliad*, other contemporary literature may be more telling for the victims of moral transgressions. Consider the events reflected in the collection *Poems from Guantanamo* (Falkoff 2007), composed of Muslim prisoners tortured by the US military. One of them reads:

those who have no courage or honor consider themselves free,

But they are slaves.

I am flying on the wings of thought,

And even in the cage, I know greater freedom (ibid., 36).

Both Améry’s words and the above poem by Muslim Dost, a Pakistani poet who reflects on subjugation, honour, and freedom when detained in Guantanamo, may offer a different conceptualisation of the ‘moral’ in MI, which accords more with the virtue-based and juridical-critical perspectives on MI rather than the psychological. Therefore, before delving into the ethical issues associated with the clinical model of MI, it is useful to briefly outline these alternative perspectives. Doing so will deepen the discussion about the epistemic limitations of MI within the trauma discourse and also set the stage for exploring the ethical concerns inherent in this model.

Bernstein’s (2015) juridical-critical perspective - named as such by Winikka-Lydon (2020) -provides a crucial framework for understanding MI. In *Torture and Dignity*, Bernstein (2015) argues that the most fitting vantage point for exploring morality is that of the victim, the one who has been harmed. This is because ‘people not principles are what get harmed, broken, violated in morally wrongful behaviors’ (ibid., 75). His concept of MI emphasizes the critical role of vulnerability, suggesting that our vulnerability stems from the potential loss of moral standing and the capacity for devastation. Bernstein views MI as an assault on dignity, particularly in violent contexts. He highlights the corporal dimension of dignity, noting that violence often targets an individual’s body. This corporeal vulnerability necessitates the protection of individuals’ dignity, their social status as humans with equal worth. Hence, social trust becomes essential. To alleviate our existential helplessness and vulnerability, we need to trust that others will recognize our dignity and refrain from causing us any harm.

Complementing Bernstein’s (2015) perspective is Wiinika-Lydon’s (2020) virtue-based discourse, which offers another valuable lens for understanding MI. Drawing on the experiences of civilians during the Bosnian War (1992-95), Wiinika-Lydon (2020) develops a theory of MI that views morality as an important aspect of our ‘interiority’, which comprises the ability to see and aspire to goodness in oneself, others and the world. Morality, he says, is ‘the ability to live into or reach toward normative, even prescriptive, horizons of behavior and relationality that provide identity and meaning to one’s life shared in common’ (Wiinika-Lydon 2019, 9). He employs moral subjectivity, the experience of a moral subject, as a framework. The moral self in this understanding is a self in a ‘field of tension’ between the pull of the Good and one’s ability - a fragile and contingent ability - to respond to that pull. This ‘field of tension’ has political aspects, desires and duties, but it also contains those morally incomprehensible experiences that can lead to failure and despair (ibid., 32). MI, therefore, is the transgression of that interiority so that the moral subject becomes unable to orient towards goodness. Although similar to the psychological discourse in that he identifies an individual element of morality, he also emphasizes moral intersubjectivity and moral orientation.

Comparing these two philosophical perspectives with the clinical model reveals significant epistemic differences in the conceptualization of MI. First, non-clinical approaches shift the focus from the perpetrators of moral transgressions to the victims. Secondly, the role of violence in the conceptualization of MI differs markedly between clinical and non-clinical models. In military contexts, violence is often framed as a neutral backdrop, with moral violations understood primarily as breaches of military rules of engagement (Frankfurt & Frazier 2016).While non-clinical models emphasize that violence profoundly influences ‘ways of seeing, ways of imagining, and ways of relating’ (Wiinika-Lydon 2019, 170), the psychological discourse pays little to no attention to the problematic nature of war violence by treating it as “natural” and “inevitable” (MacLeish 2022, 17). This omission allows MI to be considered a universal and apolitical concept, applicable even to contexts such as healthcare, where violence and killing are not inherent intentions (an issue I return to later). However, Bernstein’s (2015) and Wiinika-Lydon’s (2019, 2020) frameworks recognize the shared human condition of vulnerability to devastation amidst violence. Their models treat vulnerability as an intrinsic aspect of the human experience while framing violence not as a tragic norm but as a political and moral issue in and of itself.

This takes us to the conceptualization of the subject. Clinical discourse often envisions the individual as autonomous and self-focused, whereas alternative discourses view subjects as vulnerable and interdependent. By foregrounding relationality, different moral emotions become salient, such as trust, empathy, an or post-traumatic embitterment disorder (PTED), ‘a strong feeling of injustice and disappointment, combined with the urge to defend oneself, but with the inability to do so’ (Spaaij et al. 2021, 2).

When the prevailing discourse on MI overlooks the unique realities of refugees and instead equates their experiences with those of veterans, it marginalizes them from the process of knowledge production. This exclusion prevents the trauma discourse from adequately recognizing the experiences of those who have been wronged and addressing how they feel and act in response to the wrong. Such inequality in knowledge production is illustrated by Fricker’s (2007) concept of hermeneutic injustice, where certain groups are disadvantaged due to a lack of epistemic tools to make their experiences intelligible and communicable. In what follows, I will examine the ethical issues inherent in MI discourses. I argue that MI frameworks that do not permit victims to make moral claims leave them conceptually disadvantaged. This conceptual limitation has significant ethical implications for the victims and may render the clinical model inadequate for meeting their needs.

Concepts do not emerge from a void. The philosopher Matthew Congdon (2016) observes, they are ‘the results of discursive struggles to bring initially private experiences of suffering into a publicly recognized space of reasons’ (ibid., 818). Moral injury is no exception. As Fassin and Rechtman (2009) demonstrate in their book *The Empire of Trauma*, ‘a diagnosis can restore dignity or disgrace a person’ (ibid., 42). Hence, the ethical question arises: Can moral injury, which primarily dignifies the transgressor, also do the same for the victim? My position on this matter is negative. I will elaborate on this point further.

The conceptualization of any form of suffering is a morally significant act, but this is especially true in the case of MI, which is concerned with moral emotions. After all, moral emotions do not merely communicate the experience of a specific violation; they also request normative recognition within wider society (Congdon 2016, 816). It is often the victims, rather than the perpetrators, who assert a moral claim that an event is universally wrong, irrespective of how the offender perceives it. Congdon (2016) argues that to strip victims of their moral assertions is to inflict a ‘second injury’. He writes:

[T]he capacity to apprehend and express an experience of wrong as wrong to others is a fundamental aspect of moral life such that to take it away constitutes a significant and positive moral injury that adds to and compounds the initial injustice. When a victim’s appeals to others are met with silence, the original injury is worse than simply unaddressed. Rather, the silence constitutes a humiliating ‘second injury’ of indifference that prevents reparations, emboldens potential transgressors and alienates victims through a sense of ‘normative abandonment’ and alienation from the community (ibid., 816).

A victim’s inability to express and have their suffering acknowledged exacerbates the original harm. This silence not only intensifies the victim’s pain but also perpetuates injustice and alienation. I contend that the clinical discourse contributes to this problem for at least two main reasons: its focus on MI as an issue confined to the individual psyche and its purported political ‘neutrality’.

The clinical perspective omissions how violence and power imbalances affect embodied experiences, a critical issue for refugees, who are both minorities and highly politicised bodies. ‘Moral sensitivity’ is needed in order to attribute a moral norm to an event. If one is not aware of the morally relevant features of a situation, because of inappropriate epistemic resources, then MI will not be recognised (Congdon 2016, 828). In situations of injustice and discrimination, normative moral frameworks may not provide the tools to comprehend certain kinds of harm, as exemplified by phenomena such as sexual harassment and hate speech where the language and ‘moral sensitivity’ had to be developed to recognise those harms (ibid., 815–816). Abstracting and disregarding the lived realities of refugees - at the intersection of national, racial, and colonial politics and violence - through a purely psychological lens results in hermeneutical injustice (Fricker 2007).

Furthermore, because the psychological model treats MI as subject-generated - where moral emotions merely reflect existing beliefs without intrinsic value - it fails to provide victims of moral violations, a role refugees are more likely to assume than veterans, with a normative framework that asserts certain actions should be recognized as harmful. This is because the clinical model was designed to address the needs of military personnel, aiming to destigmatize moral failures within a military context. As a result, MI has evolved into an occupational hazard, necessitating moral education and preventive measures like leadership and resilience-building exercises to prepare workers for the demands of their roles and mitigate MI (Phelps et al. 2022). This emphasis on MI as an occupational risk factor has broadened its application to other professions, including healthcare workers and journalists. MI is now incorporated into human resources and occupational health.

This expansion of MI and its uptake by various other fields cause another ethical (and epistemic) issues. If, as Litz and Kerig (2019) argue the psychological model of MI was primarily meant to reflect the suffering of perpetrators of violence - or, even more problematically, if it is seen as a professional hazard indicative of poor individual resilience rather than a testament to the reality of violence and the precariousness faced by refugees - then its relevance and utility for refugees come into serious question.

The tendency of a concept to creep into other fields is what psychologist Haslam (2016) has termed ‘concept creep’. Concept creep refers to an expansion of a concept to phenomena that are either quantitatively (‘vertical’ creep) or qualitatively different (‘horizontal’ creep) (ibid., 2). The broadening of a concept may result in its trivialisation, de-sensitises the public and removes the distinction between the harmed and the harm-doer. Hence, in light of the exponential growth in the use of the clinical understanding of MI, it is pertinent to question whether the concept has evolved to the point of becoming irrelevant or even alienating for the refugee population. While it may be debatable whether the psychological model of MI can accurately apply to victims and perpetrators of moral violations equally, it is evident that being a refugee is not a profession. Therefore, it seems absurd to juxtapose burnout in doctors with various forms of harm and mistreatment that refugees may experience.

The expansive use of MI may unintentionally obscure the distinct experiences of traumatized refugees, who represent a particularly vulnerable group. What the anthropologist and psychiatrists Didier Fassin and Richard Rechtman (2009) critique about trauma discourse is equally applicable to MI discourse too. They assert that the concept of trauma ‘erases the uniqueness of experience,’ creating a superficial sense of familiarity while ‘true understanding is lost’ (ibid., 214–215).

## Conclusion

This paper critically investigated the epistemic and ethical issues in the application of the current clinical conceptualisation of MI to refugee populations. As psych-disciplines play a critical role in providing the epistemological tools needed to articulate human adversity, epistemic justice requires that the realities of refugees hold an authoritative voice and are treated as a legitimate site of knowledge-building. While many human experiences have moral aspects and morality is not exclusive to victims of violence, the dominant psychological discourse of MI erases the reality of refugees at the intersection of national, racialised, and colonial politics. By focusing on individual psyches and ignoring the broader political and moral aspects of war, psychological approach to MI inadvertently normalise violence perpetrated by state actors. Lastly, treating moral emotions as subjective and removing moral assertions from victims can lead to a ‘second injury’ by denying them recognition and justice.

Given these challenges, while I acknowledge the importance of capturing the moral dimensions of trauma experienced by refugees, I remain skeptical about the adequacy of the current psychological conceptualization of MI for this particular demographic. However, non-psychological frameworks, such as those from philosophy and the social sciences, provide valuable insights that merit serious consideration. These approaches are more likely to fully address the moral significance of trauma and its mental health implications, surpassing the limitations of the current notion that remains primarily rooted in military contexts. Nonetheless, a key first step to ensure that a concept of MI is relevant to refugee populations is grounding it in their lived realities, particularly the dynamics of violence, power, vulnerability, and compromised agency. Hence, collecting empirical data from specific refugee groups, rather than treating refugees as a homogeneous category, is essential for developing a more accurate understanding of the moral aspects of their experiences.
